# Comparative mitochondrial genome analysis of *Dendrolimus houi* (Lepidoptera: Lasiocampidae) and phylogenetic relationship among Lasiocampidae species

**DOI:** 10.1371/journal.pone.0232527

**Published:** 2020-05-14

**Authors:** Xiaohong Han, Huan He, Haiyan Shen, Jinhan Tang, Wanying Dong, Yufei Shi, Songqing Wu, Feiping Zhang, Guanghong Liang

**Affiliations:** 1 College of Forestry, Fujian Agriculture and Forestry University, Fuzhou, Fujian, China; 2 Key Laboratory of Integrated Pest Management in Ecological Forests, Fujian Province University, Fujian Agriculture and Forestry University, Fuzhou, Fujian, China; Onderstepoort Veterinary Institute, SOUTH AFRICA

## Abstract

*Dendrolimus houi* is one of the most common caterpillars infesting Gymnosperm trees, and widely distributed in several countries in Southeast Asia, and exists soley or coexists with several congeners and some Lasiocampidae species in various forest habitats. However, natural hybrids occasionally occur among some closely related species in the same habitat, and host preference, extreme climate stress, and geographic isolation probably lead to their uncertain taxonomic consensus. The mitochondrial DNA (mtDNA) of *D*. *houi* was extracted and sequenced by using high-throughput technology, and the mitogenome composition and characteristics were compared and analyzed of these species, then the phylogenetic relationship was constructed using the maximum likelihood method (ML) and the Bayesian method (BI) based on their 13 protein-coding genes (PCGs) dataset, which were combined and made available to download which were combined and made available to download among global Lasiocampidae species data. Mitogenome of *D*. *houi* was 15,373 bp in length, with 37 genes, including 13 PCGs, 22 tRNA genes (tRNAs) and 2 rRNA genes (rRNAs). The positions and sequences of genes were consistent with those of most known Lasiocampidae species. The nucleotide composition was highly A+T biased, accounting for ~80% of the whole mitogenome. All start codons of PCGs belonged to typical start codons ATN except for *COI* which used CGA, and most stop codons ended with standard TAA or TAG, while *COI*, *COII*, *ND4* ended with incomplete T. Only *tRNA*^*Ser (AGN)*^ lacked DHU arm, while the remainder formed a typical “clover-shaped” secondary structure. For Lasiocampidae species, their complete mitochondrial genomes ranged from 15,281 to 15,570 bp in length, and all first genes started from *trnM* in the same direction. And base composition was biased toward A and T. Finally, both two methods (ML and BI) separately revealed that the same phylogenetic relationship of *D*. spp. as ((((*D*. *punctatus* + *D*. *tabulaeformis*) + *D*. *spectabilis*) + *D*. *superans*) + (*D*. *kikuchii* of Hunan population + *D*. *houi*) as in previous research, but results were different in that *D*. *kikuchii* from a Yunnan population was included, indicating that different geographical populations of insects have differentiated. And the phylogenetic relationship among Lasiocampidae species was ((((*Dendrolimus*) + *Kunugia*) + *Euthrix*) + *Trabala*). This provides a better theoretical basis for Lasiocampidae evolution and classification for future research directions.

## Introduction

*Dendrolimus houi* Lajonquiere (Lepidoptera: Lasiocampidae), being one of the most abundant phytophagous caterpillar in southern China and some countries in Southeast Asia, voraciously feeds on about 12 species of coniferous trees, including *Cryptomeria fortunei*, *Pinus yunnanensis*, *Platycladus orientalis*, *P*. *kesiya* var.*langbianensis* and *Cupressus funebris*, causing thousands hectares of dead or dying forests, and it tends to be continuously spreading rapidly [1]. Biologically, different geographical populations of *D*. *houi* have different life cycles, host preference and adaptation to local extreme climatic factors [2,3], which might lead to population differentiation or taxonomic controversy based on previous reasearches on *D*. *kikuchii* [4] and *D*. *punctatus* [5].

Moreover, insects in the family Lasiocampidae are some of the most serious phytophagous pests worldwide, causing the host withering and rapid death, having serious impact on the ecological environment during their outbreaks [6–15]. Examples include such *D*. *pini* infesting Scots pines in Europe [16], *D*. *houi* in Yunnan, Sichuan, Fujian and Zhejiang province [2,9–10], and *D*. *punctatus* at fifteen provinces in the south of China [11]. Other speices like *D*. *tabulaeformis*, *D*. *kikuchii*, *D*. *spectabilis*, *Euthrix laeta* and *Trabala vishnou guttata* often occur in China as well [8,11], and thus face the stress of multiple and complex host and environmental factors. Consequently, they could potentially evolve in two directions: firstly, some *Dendrolimus* species may inevitably share the same host species and tend to inhabit the same forest, which might lead to taxonomically mis-discrimination [9] and hybridization [8]. (For example, *D*. *tabulaeformis* and *D*. *spectabilis* are the subspecies of *D*. *punctatus* probably due to hybridization of these three species [11,12]). Or different populations of same species might differentiated and evolved into separate species as a result of long-term adaptation to different hosts and other climatic factors [2]. (For example, *D*. *kikuchii* and *D*. *houi* is thought to have evolved from ta commmon ancestor, and evolved separately into different species as revealed by phylogenetic analysis [9]). Transcriptome analysis of antenna still showed that they still have high similarity and close phylogenetic relationship, however, they have different sex pheromone components [13], indicating somehow interspecific reproductive isolation. However, how do we identify and evaluate their ecological function and phylogenetic relationship? Fortunately, many previous studies have focused on the taxonomic relationship of Lasiocampidae, especially the taxonomic relationship within genera [5–15], but there are still some species whose taxonomic status is controversial and without a complete consensus [5–15]. Currently, some taxonomic relationships focus on the comparison with Lepidoptera [16–19], however very few reports have focused on the phylogenetic relationship among Lasiocampidae genera, so the taxonomy remains unclear.

Technically, mitochondrial genes and genomes have been generally adopted as an informative molecular marker for diverse evolutionary research of animals [5–21], and the complete and nearly complete mitogenome from hundreds of insect species has currently been determined. Generally, the length of most animal mitogenomes is 15.4–18.3 Kb [16]. It is usually a highly compact and covalently bonded closed-ring molecule composed of 37 genes, including 13 PCGs, 22 tRNAs, 2 rRNAs and a non-coding control region (D-loop region) [15–20]. In addition, animal mtDNA displays maternally inheritance, and mitogenomes have a very compact gene arrangement, high coding efficiency and overlap among some genes. Furthermore, there are no gene recombinations, translocations or inversion mutations in the genetic process. Therefore, mitogenomes have been widely used in the classification, identification, interspecific molecular evolution, population genetic and phylogenetic evolution relationships [16–23].

In this study, we obtained the complete mitogenome of *D*. *houi* and downloaded the available mtDNA data of nine species (four genera in Lasiocampidae) from the public database, containing some other congenetic species of *D*. spp., *Kunugia undans*, *E*. *laeta* and *T*. *vishnou guttata* to compare mitogenome composition and structure. Then, we reconstructed phylogenetic tree and analyzed phylogenetic relationship between *D*. *houi* and other Lasiocampidae species.

## Materials and methods

### Ethics statement

There is no endangered or protected species involved in this study, no specific permissions were required for this serious and widespread forest pest, feeding on leaves of *Cryptomeria fortunei* and causing thousands hectares of dying and dead forests. Additionally, this study is sponsored and permitted by NSFC (National Natural Science Foundation of China). We confirm that the locations are not privately owned or otherwise protected.

### Samples collection mitochondria DNA extraction

Samples of *D*. *houi* were collected in Yongtai, Fuzhou, Fujian Province, China. Mitochondrial DNA of *D*. *houi* was extracted from muscle tissue using the GENMED Mitochondrial DNA Extraction kit (Genmed Scientifics Inc., Arlington, MA, USA). Muscle tissue of *D*. *houi* was crushed under ice bath condition following instruction of Extraction kit. The quality of DNA was assessed using NanoDrop2000, qubit3.0, and 1% agarose gel electrophoresis.

### Sequencing and assembling of the mitochondrial genome

After DNA isolation, 1 μg of purified DNA was fragmented and used to construct short-insert libraries (430 bp) according to the manufacturer's instructions (Illumina, Hercules, CA, USA), and then sequenced on the Illumina Hiseq 4000 platform [24]. In order to obtain high quality clean reads, the raw reads were filtered to remove adaptors, the reads containing unknown nucleotide “N” over 10% and the duplicated sequences. Then, the clean reads were assembled into contigs using SOAPdenovo2.04 [25].

### Gene annotation and analysis

13 PCGs of *D*. *houi* mitogenome were annotated by utilizing the online program ORF, while 2 rRNAs and 22 tRNAs were annotated using the online software MITOS2 Web Server (http://mitos2.bioinf.uni-leipzig.de/index.py). The unpredicted tRNA genes used tRNAscan-SE [26] to predict secondary structure. We determined the location of each gene, and corrected the annotation based on data from the reported related species mitogenomes. Then, the genome was aligned with the Nr (non-redundant protein sequence), Swiss-Prot (a manually annotated, non-redundant protein sequence), COG (clusters of orthologous groups of proteins), GO (gene ontology) and KEGG (kyoto encyclopedia of genes and genomes) databases by BLAST v2.2.31 with a cut-off e-valve of 10^−5^ [2].

### Comparative mitogenome analyses of lasiocampidae

We downloaded available mtDNA data of nine species (four genera in Lasiocampidae) from the public database, containing *D*. *punctatus* (DP) NC_027156.1, *D*. *tabulaeformis* (DT) NC_027157.1, *D*. *spectabilis* (DSP) KJ913816.1, *D*. *superans* (DSU) NC_039841.1, *D*. *kikuchii* (DK1) NC_036347.1, *D*. *kikuchii* (DK2) MF100138.1, *K*. *undans* (KU) KX822016.1, *E*. *laeta* (EL) NC_031507.1 and *T*. *vishnou guttata* (TVG) KU884483.1) (Table 1). To obtain the information of gene loss, duplication, rearrangement, and horizontal transfer in Lasiocampidae, multiple genome alignments were conducted using Mauve software [27]. The base composition, codon usage and relative synonymous codon usage (RSCU) frequency within these 10 species of mitogenomes were analyzed by MEGA 7.0 software. The formula for calculating the composition skew in mitogenomes was as follows, AT-skew = (A-T) / (A+T), GC-skew = (G-C) / (G+C).

**Table 1:** Comparative analysis of mitogenomes and phylogenetic relationship of Lasiocampidae species information.

Family	Genus	Species	Size( bp)	GenBank accession no.	Sample sources
Lasiocampidae	*Dendrolimus*	*Dendrolimus punctatus* (DP)	15,411	NC_027156.1	-
		*Dendrolimus tabulaeformis* (DT)	15,411	NC_027157.1	-
		*Dendrolimus spectabilis* (DSP)	15,410	KJ913816.1	Taian, Shandong
		*Dendrolimus superans* (DSU)	15,417	NC_039841.1	-
		*Dendrolimus kikuchii* (DK1)	15,422	NC_036347.1	Puer, Yunnnan
		*Dendrolimus kikuchii* (DK2)	15,385	MF100138.1	Zhuzhou, Hunan
		***Dendrolimus houi*** (DH)	**15,373**	**This study**	**Fuzhou, Fujian**
	*Kunugia*	*Kunugia undans* (KU)	15,570	KX822016.1	-
	*Euthrix*	*Euthrix laeta* (EL)	15,368	NC_031507.1	Jiujiang, Jiangxi
	*Trabala*	*Trabala vishnou guttata* (TVG)	15,281	KU884483.1	Jiujiang, Jiangxi
Bombycidae	*Bombyx*	*Bombyx mori* (BM)	15,635	AB083339.1	-

### Phylogenetic analysis

The dataset of 13 PCGs, from these ten species plus *Bombyx mori* as the reference outgroup, were used to reconstructed the phylogenetic tree. Sequences blast was conducted by MAFFT of Translator X online server, the empty spaces and fuzzy sites were removed by GBlocks, and the single genes were combined to obtain the mitochondrial gene datasets, then an optimal evolution model was calculated by Modeltest 3.7 for the subsequent phylogenetic analysis. We also used RAXML 7.2.6 [28] to build a ML tree (bootstrap value is 1000), and BI analysis was carried out by using Mr Bayes 3.2.2 [29] (Markov chains were run for 1×10^5^ generations, sampling every 100 generations) to construct the phylogenetic tree of Lasiocampidae.

## Results

### Genome structure and nucleotides composition of *D*. *houi*

**Fig 1:**
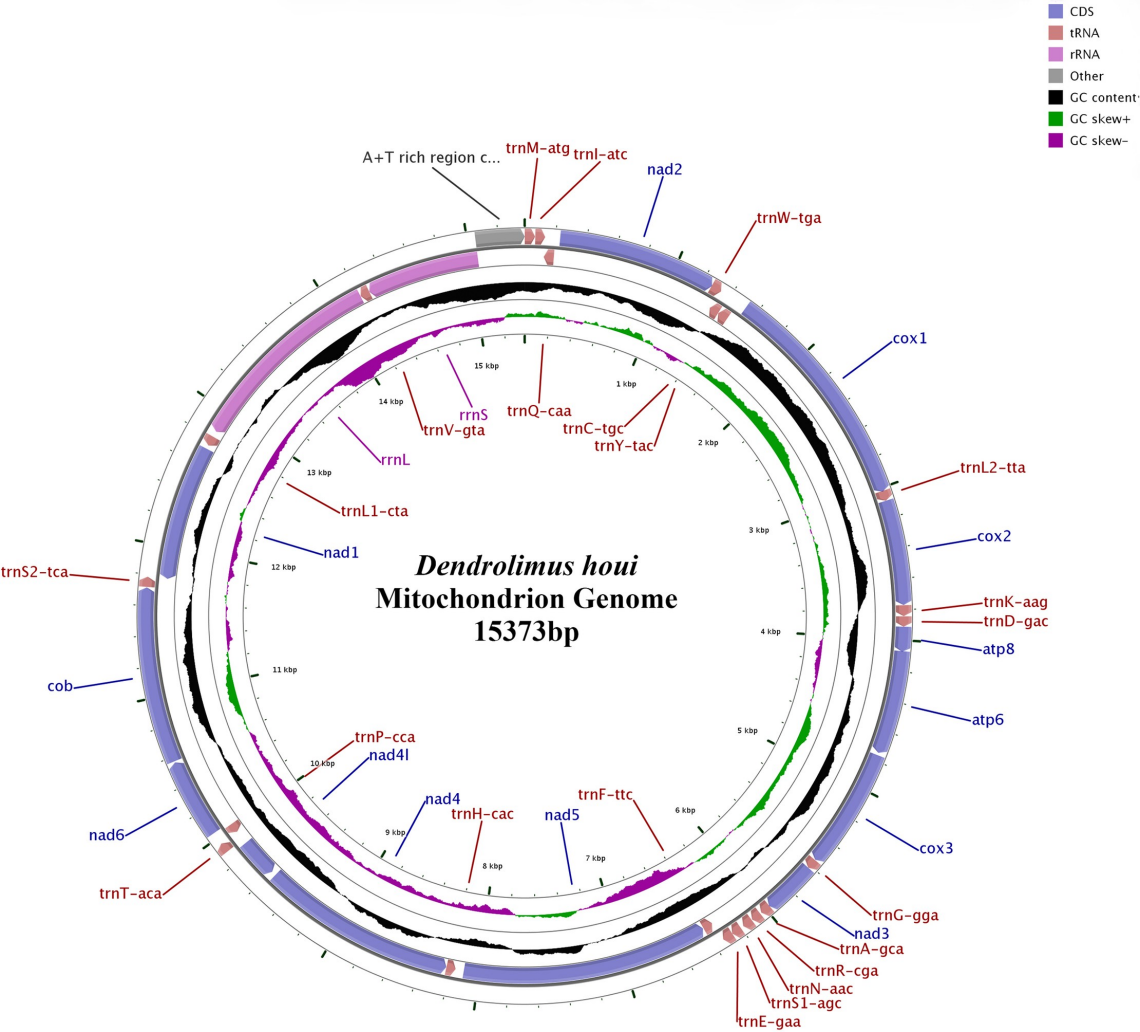
Mitochondrial genomic structure of *D*. *houi*.

The complete mitogenome of *D*. *houi* was 15,373 bp in length (Fig 1), which contained 37 genes (13 PCGs, 22 tRNAs, 2 rRNAs) and a non-coding control region (A+T-rich region) related to replication and transcription. The gene sequences were similar to that of some known related species. Among them, 23 genes were located in J-strand, including 9 PCGs (*COI*-*COIII*, *ATP6*, *ATP8*, *ND2*, *ND3*, *ND6*, *CYTB*) and 14 tRNAs, while the remaining 14 genes were located in the N-strand.

The genes in the mitogenome were closely arranged with overlapping and interval phenomena. Typically, there were 7 overlapping regions with total length of 25 bp, and the longest overlapping region was 8 bp, while there were 23 intergenic spacers with a total length of 372 bp, with the longest one 60 bp between *COI* and *tRNA*^*Tyr*^, followed by another one with 57 bp between *tRNA*^*His*^ and *ND5*. Six regions did not have overlaps or intervals (S1 Table), and the mitogenome structure of *D*. *houi* was basically similar to those of most species within Lasiocampidae.

The number of A, T, G and C in the mitogenome within *D*. *houi* were 6,321 (41.12%), 5,954 (38.73%), 1,175 (7.64%) and 1,923 (12.51%) respectively, and the content of A was the highest while G was lowest; A+T accounted for 79.85%, while G+C was 20.15%; AT skewness was 0.0299 and GC skewness was -0.2417 (S2 Table). Furthermore, the A+T content at codon site 2 of PCGs (79.70%) was slightly higher than that at the first site (79.43%), while the A+T content at site 3 was the highest (80.43%). The non-coding region was especially A+T -rich, with the total content was as high as 91.85%. AT skewness was negative, which indicated that the content of T was higher than A.

The PCGs sequence was 11,091 bp, accounting for 72.15% of the complete mitogenome. A, T, G, C bases were 3,749 (33.8%), 4,924 (44.4%), 1,239 (11.17%), 1,179 (10.63%), respectively. The A+T content was 78.20%, which was 1.65% lower than that of the whole mitogenome. The complementary G+C content accounted for 21.80%, which was different from the whole mitogenome, with the highest T content and the lowest C content. AT skewness was -0.1356, GC skewness was 0.0248. A total of 4,617 codons (excluding stop codon) were encoded by 13 PCGs within the mitogenome. The most frequently encoded amino acids were Leu (13.26%), Ile (10.83%), Asn (10.66%), Phe (9.75%), and Lys (9.18%) respectively. The frequency of relative synonymous codons showed that the mitogenome of *D*. *houi* had obvious bias towards A and T, for example when it encoded the same amino acid, it was preferred to use UUU(Phe), UUA(Leu), AUU(Ile), AUA(Met), GUA(Val), UCU(Ser), CCU(Pro), ACU(Thr), GCA(Ala), UAU(Tyr), CAU(His), CAA(Gln), AAU(Asn), AAA(Lys), GAU(Asp), GAA(Glu), UGU(Cys), UGG(Trp), AGA(Arg), GCA(Gly). Most bases within each codon were composed of A and U, and the RSCU values of each codon varied significantly, which indicated that the codon frequency was biased in the mitogenome of *D*. *houi* (S1 Fig).

Mitogenome of *D*. *houi* contained 22 tRNA genes with total length 1,475 bp, and the sequences lengths were between 64–71 bp. Twenty-two secondary structures of tRNA genes were basically similar to those of other species of Lepidoptera, and twenty-one of them had typical clover structures consisting of amino acid receiving arms, DHU arm, anticodon arm, variable ring and TΨC arm. Only *tRNA*^*Ser (AGN)*^ could not form a complete structure due to absence of dihydrouridine (DHU) arm. Based on the predicted 22 tRNA secondary structures, there were also nonstandard T-C, G-T and T-T in additional to mismatches except for the standard A-T and G-C matches (S2 Fig). Two rRNAs (*rRNAL* and *rRNAS*) of mitogenome between *tRNA*^*Leu (CUN)*^ and control region (CR) were encoded by L-strand and separated by *tRNA*^*Val*^ gene. The length of *rRNAL* was 1,365 bp, A+T content was 83.51%, AT skewness value was -0.0470. The length of *rRNAS* was 776 bp, A+T content was 85.70%, AT skewness value was 0.0345.

The A+T -rich region was the main non-coding region of mitogenomes, located between *rRNAS* and *tRNA*^*Met*^ genes, with a total length of 319 bp. The A+T content was 91.85%, which was significantly higher than other genes of the mitogenome (S2 Table). There were also typical structural features of Lepidopteran mitogenomes in the CR. The results also showed that there was a 14 bp poly-T stretch with motif ATAGA that was 15,075–15,093 bp downstream of *rRNAS* gen, and 4 microsatellite-like repeat sequences containing AT, AAT and AAT in this region. Additionally, there were also multiple poly-T stretch in this region (S3 Fig) except for the poly-T stretch at the beginning of replication.

### Comparative analysis of mitogenomes of lasiocampidae species

**Fig 2:**
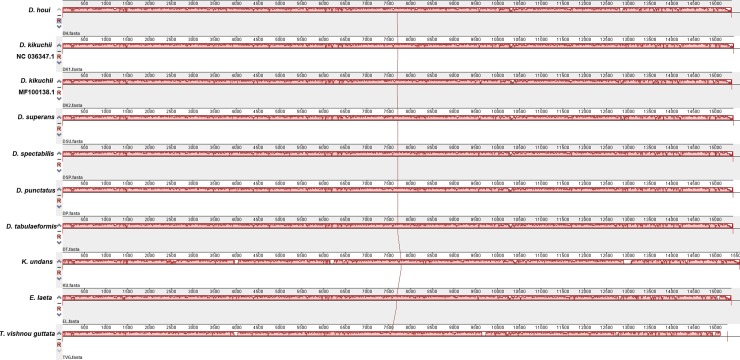
Mauve (multiple alignment of sequence with rearrangements) alignment of mitogenomes of ten species. The *D*. *houi* mitogenome was shown at the top as the reference genome. Within each of the alignments, local collinear blocks are represented by blocks of the same color connected by lines.

**Fig 3:**
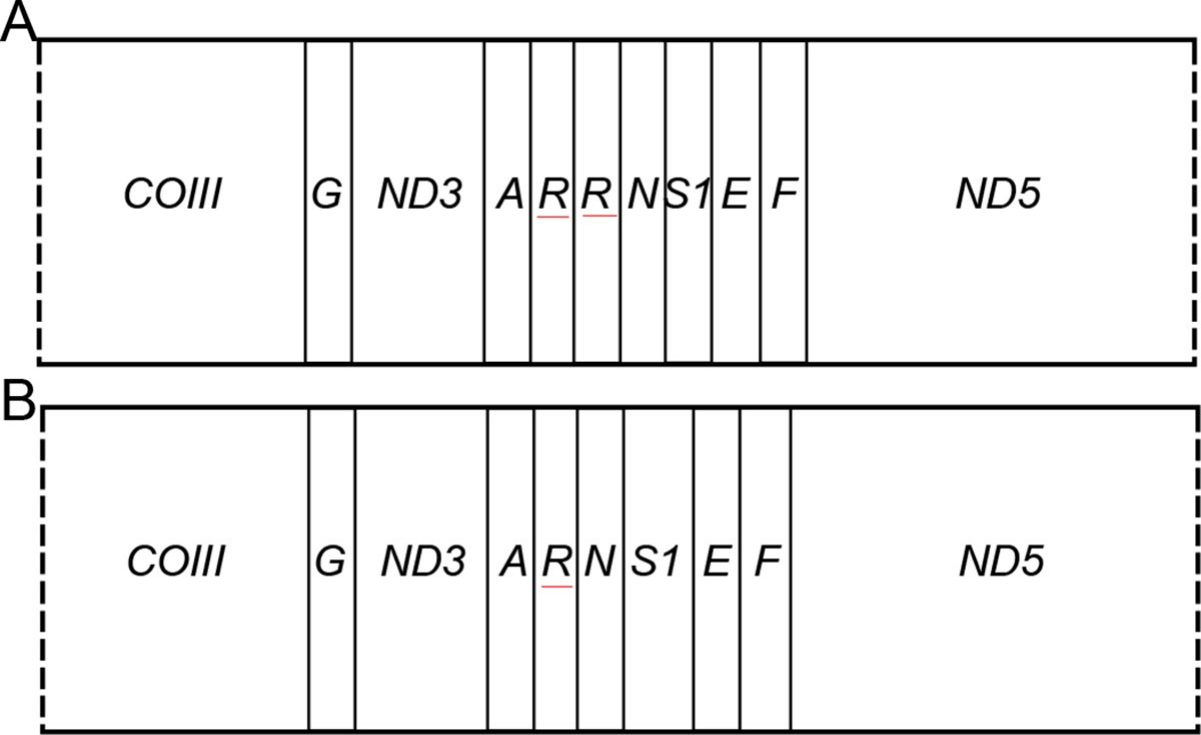
Arrangement of mtDNA genes in Lasiocampidae species. A: Arrangement of *Kunugia undans* mtDNA, B: Arrangement of Lasiocampidae mtDNA.

Complete genome alignment using Mauve software was done for 10 species of Lasiocampidae (*Dendrolimus* spp., 1 *Kunugia*, 1 *Euthrix* and 1 *Trabala*) (Table 1, Fig 2). Generally, most of the genes within these ten species maintain a consistent position and direction, and no rearrangement or inversion events were found in the locally-collinear blocks (LCBs). Interestingly, there were two specific *tRNA*^*Arg*^ (Fig 3A) within the mitogemone of *K*. *undans*, which was absent among Lasiocampidae (Fig 3B).

The complete mitochondrial genomes of Lasiocampidae ranged from 15,281 to 15,570 bp in length, TVG was 15,281 bp and KU was 15,570 bp (Table 2). The first genes of these species all started from *trnM*, and had same direction. Moreover, there was base composition bias toward A and T in these mitogenomes. The A+T content of these mitogenomes ranged from 78.64% to 80.87%, KU was 78.64% and TVG was 80.87% (Table 3).

**Table 2:** Genome organization of ten species.

Gene	D	DP	DT	DSP	DSU	DK1	DK2	DH	KU	EL	TVG
*tRNAMet*	F	1–67 (-)	1–67 (-)	1–67 (-)	1–68 (-)	1–67 (-)	1–67 (-)	1–67 (-)	1–68 (-)	1–68 (-)	1–64 (-)
*tRNAIle*	F	71–134 (3)	71–134 (3)	71–134 (3)	72–135 (3)	71–134 (3)	71–134 (3)	69–132 (1)	72–135 (3)	72–135 (3)	70–133 (5)
*tRNAGln*	R	132–200 (-3)	132–200 (-3)	132–200 (-3)	133–201 (-3)	132–200 (-3)	132–200 (-3)	130–198 (-3)	136–205 (0)	133–201 (-3)	131–199 (-3)
*ND2*	F	259–1266 (58)	259–1266 (58)	259–1266 (58)	256–1266 (54)	259–1266 (58)	250–1263 (49)	230–1264 (31)	263–1276 (57)	272–1276 (70)	254–1261 (54)
*tRNATrp*	F	1265–1333 (-2)	1265–1333 (-2)	1265–1333 (-2)	1265–1336 (-2)	1265–1333 (-2)	1263–1333 (-1)	1263–1332 (-2)	1275–1344 (-2)	1275–1342 (-2)	1260–1327 (-2)
*tRNACys*	R	1326–1391 (-8)	1326–1391 (-8)	1326–1391 (-8)	1329–1394 (-8)	1326–1391 (-8)	1334–1391 (0)	1325–1391 (-8)	1337–1402 (-8)	1335–1400 (-8)	1320–1385 (-8)
*tRNATyr*	R	1392–1459 (0)	1392–1459 (0)	1392–1459 (0)	1395–1460 (0)	1392–1459 (0)	1392–1459 (0)	1393–1463 (1)	1412–1479 (9)	1418–1483 (17)	1386–1448 (0)
*COI*	F	1487–3017 (27)	1494–3024 (34)	1485–3015 (25)	1493–3023 (32)	1494–3024 (34)	1500–3030 (-10)	1524–3026 (60)	1500–3057 (20)	1490–3020 (6)	1464–2994 (15)
*tRNALeu(UUN)*	F	3018–3084 (0)	3025–3091 (0)	3016–3082 (0)	3024–3090 (0)	3025–3091 (0)	3031–3097 (0)	3022–3088 (-5)	3058–3125 (0)	3021–3087 (0)	2995–3061 (0)
*COII*	F	3085–3766 (0)	3092–3773 (0)	3083–3764 (0)	3091–3772 (0)	3092–3773 (0)	3098–3779 (0)	3089–3772 (0)	3125–3806 (-1)	3088–3769 (0)	3062–3743 (0)
*tRNALys*	F	3767–3837 (0)	3774–3844 (0)	3765–3835 (0)	3773–3843 (0)	3774–3844 (0)	3780–3850 (0)	3774–3844 (1)	3807–3877 (0)	3770–3840 (0)	3744–3814 (0)
*tRNAAsp*	F	3841–3908 (3)	3848–3915 (3)	3839–3906 (3)	3847–3914 (3)	3848–3915 (3)	3851–3918 (0)	3846–3912 (1)	3879–3947 (1)	3846–3912 (5)	3815–3879 (0)
*ATP8*	F	3909–4070 (0)	3916–4074 (0)	3907–4065 (0)	3915–4076 (0)	3916–4074 (0)	3919–4080 (0)	3913–4074 (0)	3948–4109 (0)	3913–4077 (0)	3880–4044 (0)
*ATP6*	F	4064–4741 (-7)	4068–4745 (-7)	4059–4736 (-7)	4070–4747 (-7)	4068–4745 (-7)	4074–4751 (-7)	4071–4745 (-4)	4103–4780 (-7)	4071–4748 (-7)	4038–4715 (-7)
*COIII*	F	4757–5545 (15)	4761–5549 (15)	4748–5536 (11)	4760–5548 (12)	4761–5549 (15)	4758–5546 (6)	4762–5550 (16)	4787–5575 (6)	4753–5541 (4)	4735–5523 (19)
*tRNAGly*	F	5548–5613 (2)	5552–5617 (2)	5539–5604 (2)	5551–5616 (2)	5552–5617 (2)	5549–5615 (2)	5553–5618 (2)	5578–5644 (2)	5544–5609 (2)	5526–5591 (2)
*ND3*	F	5614–5967 (0)	5618–5971 (0)	5605–5958 (0)	5617–5970 (0)	5618–5971 (0)	5616–5969 (0)	5619–5972 (0)	5645–5998 (0)	5610–5963 (0)	5592–5945 (0)
*tRNAAla*	F	5967–6033 (-1)	5971–6037 (-1)	5958–6024 (-1)	5975–6041 (4)	5971–6037 (-1)	5972–6037 (2)	5971–6038 (-2)	6003–6070 (4)	5974–6039 (10)	5944–6010 (-2)
*TRNAArg*	F	6049–6112 (15)	6053–6116 (15)	6045–6108 (20)	6055–6118 (13)	6058–6121 (20)	6064–6128 (6)	6048–6112 (9)	6084-6147/6175-6241(13/27)	6052–6120 (12)	6024–6088 (13)
*tRNAsn*	F	6117–6182 (4)	6121-6186(4)	6113–6178 (4)	6120–6185 (1)	6126–6191 (4)	6150–6216 (21)	6117–6183 (4)	6242–6308 (0)	6122–6186 (1)	6094–6159 (5)
*TRNASer(AGN)*	F	6194–6261 (11)	6198–6265 (11)	6197–6264 (18)	6202–6269 (16)	6211–6276 (19)	6232–6297 (15)	6204–6269 (20)	6308–6375 (-1)	6192–6257 (5)	6160–6220 (0)
*tRNAGlu*	F	6261–6326 (-1)	6265–6329 (-1)	6264–6328 (-1)	6269–6333 (-1)	6277–6342 (0)	6298–6361 (0)	6270–6338 (0)	6376–6440 (0)	6258–6323 (0)	6221–6286 (0)
*tRNAPhe*	R	6331–6396 (4)	6334–6399 (4)	6337–6402 (8)	6346–6412 (12)	6351–6416 (8)	6375–6441 (13)	6347–6413 (8)	6471–6537 (30)	6328–6395 (4)	6285–6349 (-2)
*ND5*	R	6399–8141 (2)	6402–8144 (2)	6406–8148 (3)	6416–8158 (3)	6420–8162 (3)	6446–8185 (4)	6418–8103 (4)	6538–8275 (0)	6394–8133 (-2)	6349–8088 (-1)
*tRNAHis*	R	8142–8209 (0)	8145–8212 (0)	8149–8216 (0)	8159–8226 (0)	8163–8230 (0)	8186–8250 (0)	8161–8224 (57)	8276–8343 (0)	8134–8197 (0)	8089–8154 (0)
*ND4*	R	8210–9548 (0)	8213–9551 (0)	8217–9555 (0)	8227–9565 (0)	8231–9569 (0)	8251–9592 (0)	8226–9563 (1)	8348–9682 (4)	8197–9537 (-1)	8155–9493 (0)
*ND4L*	R	9573–9866 (24)	9576–9869 (24)	9579–9872 (23)	9604–9897 (38)	9593–9880 (23)	9625–9918 (2)	9595–9861 (31)	9688–9981 (5)	9562–9855 (24)	9503–9796 (9)
*tRNAThr*	F	9874–9937 (7)	9877–9940 (7)	9880–9943 (7)	9905–9970 (7)	9894–9957 (13)	9923–9987 (4)	9893–9957 (31)	9986–10050 (4)	9860–9923 (4)	9801–9864 (4)
*tRNAPro*	R	9938–10002 (0)	9941–10005 (0)	9944–10008 (0)	9971–10035 (0)	9958–10022 (0)	9988–10052 (0)	9958–10022 (0)	10051–10115 (0)	9924–9988 (0)	9865–9929 (0)
*ND6*	F	10011–10541 (8)	10014–10544 (8)	10017–10547 (8)	10044–10574 (8)	10043–10561 (20)	10061–10591 (8)	10040–10561 (17)	10124–10654 (8)	9997–10524 (8)	9941–10465 (11)
*CytB*	F	10546–11694 (4)	10549–11697 (4)	10552–11700 (4)	10579–11727 (4)	10569–11714 (7)	10595–11743 (3)	10570–11715 (8)	10662–11807 (7)	10537–11685 (12)	10478–11626 (12)
*TRNASer(UCN)*	F	11698–11764 (3)	11701–11767 (3)	11704–11769 (3)	11731–11797 (3)	11718–11783 (3)	11742–11807 (-2)	11719–11784 (3)	11809–11875 (1)	11688–11754 (2)	11625–11692 (-2)
*ND1*	R	11764–12717 (-1)	11767–12720 (-1)	11769–12722 (-1)	11797–12750 (-1)	11783–12736 (-1)	11807–12760 (-1)	11784–12713 (-1)	11869–12825 (-7)	11753–12709 (-2)	11691–12647 (-2)
*tRNALeu(CUN)*	R	12719–12786 (1)	12722–12789 (1)	12724–12791 (1)	12752–12820 (1)	12738–12805 (1)	12762–12832 (1)	12739–12806 (25)	12827–12892 (1)	12711-12780(1)	12648–12718 (0)
*rRNAL*	R	12787–14247 (0)	12790–14248 (0)	12792–14245 (0)	12821–14253 (0)	12907–14263 (101)	12831–14214 (-2)	12847–14211 (40)	12893–14406 (0)	12766–14155 (-15)	12704–14057 (-15)
*tRNAVal*	R	14248–14312 (0)	14249–14313 (0)	14246–14311 (0)	14254–14319 (0)	14260–14325 (-4)	14216–14280 (1)	14211–14278 (1)	14407–14471 (0)	14155–14220 (-1)	14056–14120 (-2)
*rRNAS*	R	14313–15091 (0)	14314–15091 (0)	14312–15090 (0)	14320–15101 (0)	14326–15102 (0)	14282–15062 (1)	14279–15054 (0)	14472–15253 (0)	14221–14996 (0)	14121–14898 (0)
*Control region*	F	15092–15411	15092–15411	15091–15410	15102–15417	15103–15422	15063–15382	15055–15373	15254–15570	14997–15368	14936–15281

The value in parentheses: the positive number indicates interval base pairs between genes, while the negative indicates the overlapping base pairs between genes. “D” means that “Direction”.

**Table 3:** A+T content of Lasiocampidae.

Species	Size (bp)	A+T content (%)			
		Whole genome	1st codon positions	2nd codon positions	3rd codon positions
**DP**	15411	79.5	80.5	75.4	81.7
**DT**	15411	79.5	77.7	79.8	82.3
**DSP**	15410	79.38	80.42	78.45	79.26
**DSU**	15417	80.13	82.72	79.47	78.21
**DK1**	15422	79.20	76.62	82.01	78.97
**DK2**	15383	78.70	78.35	83.01	74.74
**DH**	15373	79.85	79.43	79.70	80.43
**KU**	15570	78.64	79.23	81.43	75.26
**EL**	15368	80.19	83.12	77.57	79.89
**TVG**	15281	80.87	81.94	82.69	77.97

Start and stop codon usage is an important characteristic in the annotation of PCGs. We compared the start and stop codons across the 10 species of Lasiocampidae (Table 4). The start codon of these ten species was the typical ATN codons except for *COI* (CGA), and there were some different start codons on genes of *ND2*, *ATP8*, *ND3*, *ND5*, *ND4*, *ND4L* and *ND1*. The PCGs were used the same stop codon except for *ND3*, *ND5*, *ND4* and *ND4L*. Furthermore, we compared codon usage and RSCU of ten available Lasiocampidae mitogenomes (S3 Table). Examination of these ten individual Lasiocampidae mitogenomes showed that Leu2 (UUA), Ser2 (UCU), Ala (GCU), Ser1 (AGA) were the four most frequent relatively synonymous codons.

**Table 4:** Start codon and stop codon of 13 PCGs in Lasiocampidae species.

Species	*ND2*	*COI*	*COII*	*ATP8*	*ATP6*	*COIII*	*ND3*	*ND5*	*ND4*	*ND4L*	*ND6*	*CytB*	*ND1*
**DP**	ATT/TAA	CGA/T	ATA/T	ATT/TAA	ATG/TAA	ATG/TAA	ATG/TAA	ATT/TAA	ATG/T	ATG/TAA	ATA/TAA	ATG/TAA	ATG/TAA
**DT**	ATT/TAA	CGA/T	ATA/T	ATT/TAA	ATG/TAA	ATG/TAA	ATG/TAA	ATT/TAA	ATG/T	ATG/TAA	ATA/TAA	ATG/TAA	ATG/TAA
**DSP**	ATT/TAA	CGA/T	ATA/T	ATC/TAA	ATG/TAA	ATG/TAA	ATC/TAA	ATT/TAA	ATG/T	ATG/TAA	ATA/TAA	ATG/TAA	ATG/TAA
**DSU**	ATC/TAA	CGA/T	ATA/T	ATT/TAA	ATG/TAA	ATG/TAA	ATC/TAA	ATT/TAA	ATG/T	ATG/TAA	ATA/TAA	ATG/TAA	ATG/TAA
**DK1**	ATT/TAA	CGA/T	ATA/T	ATC/TAA	ATG/TAA	ATG/TAA	ATT/TAA	ATT/TAA	ATG/T	ATT/TAA	ATA/TAA	ATA/TAA	ATG/TAA
**DK2**	ATT/TAA	CGA/T	ATA/T	ATA/TAA	ATG/TAA	ATG/TAA	ATT/TAA	ATA/TAA	ATG/T	ATG/TAA	ATA/TAA	ATG/TAA	ATG/TAA
**DH**	ATA/TAA	CGA/T	ATA/T	ATT/TAA	ATG/TAA	ATG/TAA	ATT/TAG	ATA/TAA	ATG/T	ATG/TAA	ATA/TAA	ATG/TAA	ATG/TAA
**KU**	ATT/TAA	CGA/T	ATA/T	ATC/TAA	ATG/TAA	ATG/TAA	ATC/TAA	ATT/T	ATG/TAG	ATG/TAG	ATA/TAA	ATG/TAA	ATG/TAA
**EL**	ATA/TAA	CGA/T	ATA/T	ATC/TAA	ATG/TAA	ATG/TAA	ATT/TAA	ATT/TAA	ATG/TAA	ATA/TAA	ATA/TAA	ATG/TAA	ATG/TAA
**TVG**	ATT/TAA	CGA/T	ATA/T	ATC/TAA	ATG/TAA	ATG/TAA	ATT/TAA	ATT/TAA	ATG/T	ATG/TAA	ATA/TAA	ATG/TAA	ATT/TAA

### Phylogenetic relationship

**Fig 4:**
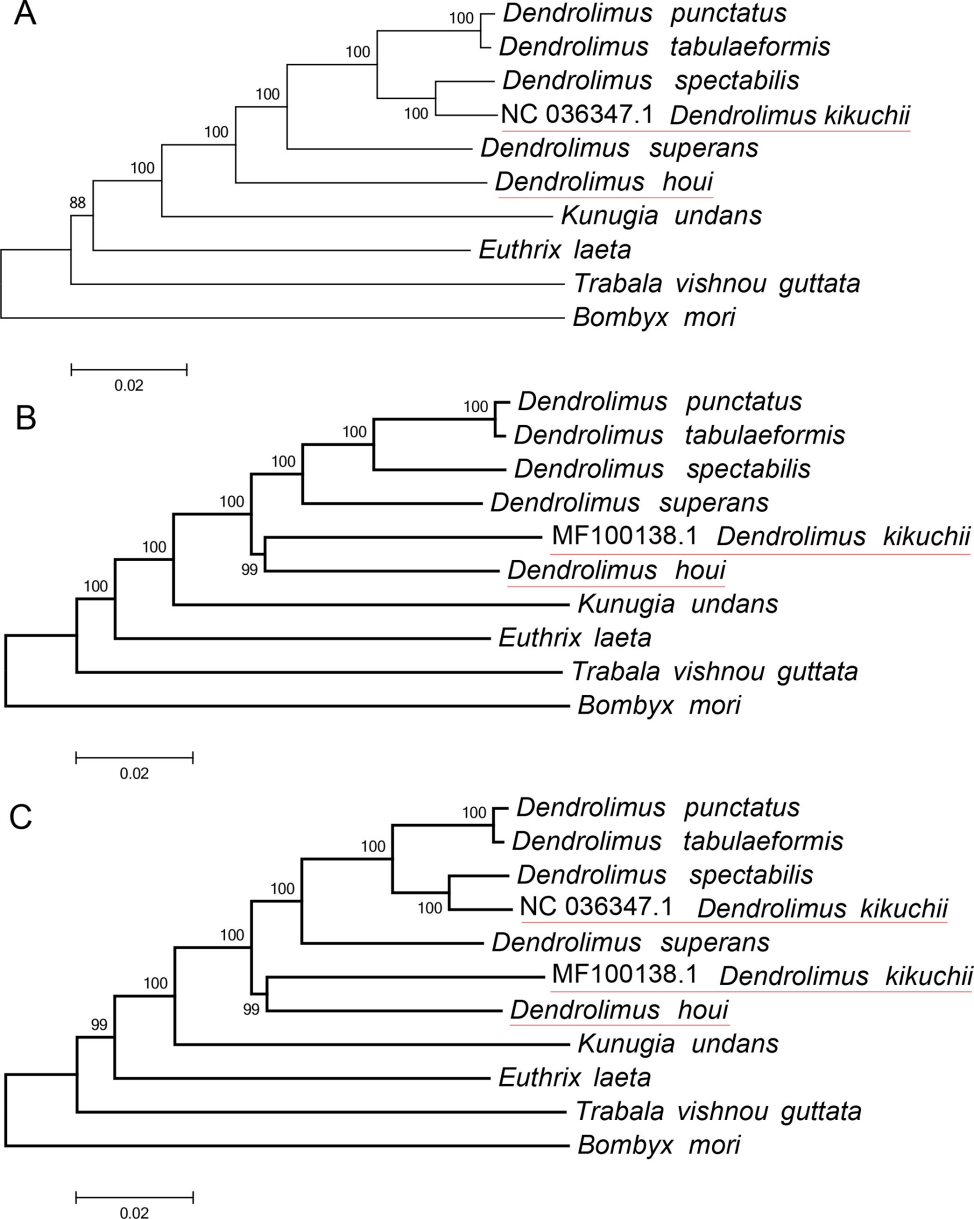
Lasiocampidae phylogenetic tree. A: using the data of DK1, B: using the data of DK2, C: using the data of DK1 and DK2.

The result showed that the best model of ML and BI trees was GTR+I+G, where 1nL value was -163,261.5358, AIC value was 326,791.0716, and ΔAIC value was 0. Two types of phylogenetic trees were constructed by using the 13 PCGs dataset having the same structure as Fig 4, which indicate that both methods (ML and BI) and results were consistent and reliable. The phylogenetic relationship among four genera was ((((*Dendrolimus*) + *Kunugia*) + *Euthrix*) + *Trabala*). Interestingly, we found two different phylogenetic trees can be constructed by using two groups data of *D*. *kikuchii*. When we use the data of DK1 to construct tree, the relationship was ((((*D*. *punctatus* + *D*. *tabulaeformis*) + (*D*. *spectabilis* + DK1)) + *D*. *superans*) + *D*. *houi*) (Fig 4A). However, the relationship have changed as ((((*D*. *punctatus* + *D*. *tabulaeformis*) + *D*. *spectabilis*) + *D*. *superans*)+ (DK2 + *D*. *houi*) (Fig 4B) by using the data of DK2. If two groups of *D*. *kikuchii* data were used simultaneously to construct phylogenetic tree, their phylogenetic relationship demonstrated that those two groups of DK data have different genetic relationship with *D*. *houi* (Fig 4C).

## Discussion

In this study, the complete mitogenome of *D*. *houi* was obtained, with a total length of 15,373 bp. The mitogenome of *D*. *houi* had similar structural composition and gene arrangement, which indicated that the mitogenome was stable and suitable for the study of phylogenetic relationships. Technically, we obtained the mitogenome by using high-throughput sequencing which was different from previous research [30–33]. The traditional mitogenome sequencing is mainly Sanger sequencing method based on PCR amplification products [34]. However, this method requires primer information of each segment of the genome, and the experimental process is complex, time-consuming and laborious. With the development of next generation sequencing technology (NGS) [33–35], the use of high-throughput sequencing technology has provided great convenience for the rapid acquisition of mitogenome [35–37], and the sequencing cost has also been significantly reduced in recent years, which provides an alternative choice for the sequencing of small genomes such as animal mitochondria [37].

Mitogenomes are more comprehensive and accurate in species identification and phylogeny, avoiding artificial biases caused by blindness of a single gene [38–43], and even the differences among species can be determined based on genes structure and arrangement, which has higher reliability [31,43]. Methodologically, using the 13 PCGs in the sequencing genome was superior in obtaining reliable results compared with clustering analysis by using individual genes [44–50], while both two clustering trees by using both methods were consistent, and the phylogenic relationship among 4 genera in Lasiocampidae was consistent with some previous studies [15,50–52]. In this study, the mitogenome total lengths of *D*. *houi* from two geographical populations (Yongtai and Jingdong [10]) were various, which may be caused by individual differences of samples. Furthermore, two original data of *D*. *kikuchii* were used to compare mitogenome structures and construct phylogenetic trees. Interestingly, we obtained the different phylogenetic trees concerning the relationship between *D*. *houi* and *D*. *kikuchii*. The phylogenetic relationship between *D*. *kikuchii* and *D*. *spectabilis* was closer than that between *D*. *kikuchii* and *D*. *houi* by using DK1 data (Fig 4A), which was different from previous research [7–8,10]. However, we obtained the same result as previous reports [10] by utilizing DK2 data (Fig 4B), and show *D*. *houi* and *D*. *kikuchii* have a close relationship (Fig 4B) and evolved earlier than the other four pine caterpillar species. Obviously, some populations of *D*. *kikuchii* from different locations can not be clustered as one group (Fig 4C) whereas DK1 and DK2 data were clustered simultaneously, indicating differentiation occurred due to geographical isolation [4].

These differences were thought to be mostly caused by individual differences or geographic population variation, because the samples of DK1 and DK2 were originally collected originally from Yunnan and Hunan Province respectively [6,14], and some previous studies have shown that insects have generated genetic variation after long-term living in different geographical habitats or from feeding on different hosts [4,5,53]. The degree of genetic differentiation and gene exchange within and between populations have an impact on the geographical populations’ genetic diversity of insects. However, even now, little is known about the different geographical populations genetic diversity of *D*. *houi*. Effective molecular markers should be used to prove whether there is differentiation among different populations, or to better determine the genetic relationship and variation at the genus level.

In this study, all species looked at have the start codon CGA with *COI* gene, which were reported in previous research [11–19,54,55]. Theoretically, it is considered a typical trait due to it being high conservation in Lepidoptera specicies. Similarly, the stop codon ended with TAA in the majority of PCGs, which was also consistent with the results of most Lepidoptera species [6–10]. Currently, there was only one *trnR* within the mitochondrial structure of *D*. *houi*, which is similar to most species of Lasiocampidae speices, and different from that of *K*.*undans* with two *trnR*s. Therefore, we presume that the structure of two *trnR*s is unique in *Kunugia* spp., which requires further clarification because more species’ genomes are not available at this time.

Currently, the sequencing of animal mitogenomes is rapidly increasing, but the data of insect mitogenomes are obviously still insufficient. Actually, several species of pine caterpillars periodically occur and outbreak [1–3,9,10], endangering local coniferous forests in China [56] under the reasonable climate. Their phylogenetic relationship might be complicated because of homogenization or hybrid of congeneric species. Therefore, the acquisition of the complete mitogenome of *D*. *houi* and comparison to Lasiocampidae species may provide more genetic varieties for future research.

## Supporting information

S1 Fig The relative synonymous codon usage frequency of the mitogenome of *Dendrolimus houi* (RSCU). (TIF)

S2 Fig Secondary structure of mitogenome tRNA of *Dendrolimus houi*. (TIF)

S3 Fig Structure of A +T-rich region of *Dendrolimus houi*. (TIF)

S1 Table Composition and characteristics of mitogenome of *Dendrolimus houi*. (XLS)

S2 Table Mitogenomic nucleotides composition of *Dendrolimus houi*. (XLS)

S3 Table The relative synonymous codon usage frequency of the mitogenome of ten Lasiocampidae species (RSCU). (XLS)

S1 File Mitochondrial genome of *Dendrolimus houi*. (FA)

## References

[1] Liang GH, Lin HY, Lu CD, Han XH, Hua Y, Huang XJ, et al Morphology and biology of seven parasitic flies of *Dendrolimus houi* in China. Plant Protection. 2018; 44(06):177–184. Available from: https://www.cnki.net/kcms/doi/10.16688/j.zwbh.2018194.html

[2] Han XH, Lu CD, Geib SM, Zheng JX, Wu SQ, Zhang FP, et al (2019). Characterization of *Dendrolimus houi* Lajonquiere (Lepidoptera: Lasiocampidae) transcriptome across all life stages. Insects. 2019; 10(12): 442 10.3390/insects10120442 PMC6956129 31835398

[3] Lin HY, Fu LQ, Lin JH, Hua Y, Han XH, Zheng JX, et al Main species of parasitic natural enemy insects within *Dendrolimus houi* (Lajonquiere) in the forest of *Cryptomeria fortunei* (Hooibrenk). Chinese Journal of Biological Control. 2017; 33(06):842–848. Available from: http://www.zgswfz.com.cn/CN/10.16409/j.cnki.2095-039x.2017.06.018

[4] Men Q, Xue G, Mu D, Hu Q, Huang M. Mitochondrial DNA markers reveal high genetic diversity and strong genetic differentiation in populations of *Dendrolimus kikuchii* Matsumura (Lepidoptera: Lasiocampidae). PLoS One. 2017; 12(6), e0179706 10.1371/journal.pone.0179706 28662066 PMC5491029

[5] Li J, Jin Q, Zhu GP, Jiang C, Zhang AB. Phylogeography of *Dendrolimus punctatus* (Lepidoptera: Lasiocampidae): Population differentiation and last glacial maximum survival. Ecology and Evolution. 2019; 9(21). 10.1002/ece3.5278 PMC6635939 31346417

[6] Wu YH, Gu XS, Xue J, Wang X. The complete mitochondrial genome of *Dendrolimus kikuchii* (Lepidoptera: Lasiocampidae). Mitochondrial DNA Part B. 2017; 2(2): 536–537. 10.1080/23802359.2017.1365654 PMC7799951 33473890

[7] Dai QY, Gao Q, Wu CS, Chesters D, Zhu CD, Zhang AB. Phylogenetic Reconstruction and DNA Barcoding for Closely Related Pine Moth Species (*Dendrolimus*) in China with Multiple Gene Markers. PLoS One. 2012; 7(4): e32544 10.1371/journal.pone.0032544 22509245 PMC3317921

[8] Qin J, Zhang YZ, Zhou X, Kong XB, Wei SJ, Ward RD, et al Mitochondrial phylogenomics and genetic relationships of closely related pine moth (Lasiocampidae: *Dendrolimus*) species in China, using whole mitochondrial genomes. BMC Genomics. 2015; 16(1):428 10.1186/s12864-015-1566-5 26040695 PMC4455531

[9] Kong XB, Zhang Z, Zhao CH, et al Female sex pheromone of the Yunnan pine caterpillar moth Dendrolimus houi: First (E, Z)—isomers in pheromone components of *Dendrolimus* spp. Journal of Chemical Ecology, 2007; 33(7): 1316–1327. 10.1007/s10886-007-9313-2 17541690

[10] Wang Y, Kong XB, Zhang SF, Liu F, Zhang Z, Yan SC. Sequencing and Analysis of Complete Mitochondrial Genome of *Dendrolimus houi* Lajonquiere (Lepidoptera: Lasiocampidae). Forest Research. 2019; 32(5):11–20. Available from: http://www.lykxyj.com/cn/article/doi/10.13275/j.cnki.lykxyj.2019.05.002

[11] Zhang AB, Kong XB, Li DM, Liu YQ. DNA fingerprinting evidence for the phylogenetic relationship of eight species and subspecies of *Dendrolimus* (Lepidoptera: Lasiocampidae) in China. Acta Entomologica Sinica. 2004; 47(2):236–242. Available from: http://www.insect.org.cn/CN/Y2004/V47/I2/236

[12] Zhao QS, Wu WB, Lu GP, Yuan X, Li SK, Jiang JC. Hybridization experiments with two species of *Dendrolimus*. Acta Entomologica Sinica. 1992; 35(1):28–32. Available from: http://www.insect.org.cn/CN/Y1992/V35/I1/28

[13] Sun FC, Kong XB, Zhang SF, Wang HB, Zhang Z, Liu F. Geographic variation of sex pheromones in three populations of *Dendrolimus kikuchii* (Lepidoptera: Lasiocampidae). Forest Research. 2017; 30(06):993–998. Available from: http://www.lykxyj.com/cn/article/doi/10.13275/j.cnki.lykxyj.2017.06.015

[14] Liu JH, Fu JQ, Yang LY, Jia PF. Complete mitochondrial genome of Simao pine caterpillar moth, *Dendrolimus kikuchii* (Lepidoptera: Lasiocampidae) and the related phylogenetic analysis. Mitochondrial DNA Part B. 2017; 2(2): 611–612. 10.1080/23802359.2017.1372725 PMC7800353 33490468

[15] Kim MJ, Kim JS, Kim SS, Kim SR, Kim I. Complete mitochondrial genome of the pine moth, *Dendrolimus spectabilis*, (Lepidoptera: Lasiocampidae). Mitochondrial DNA Part B. 2016; 1(1):180–181. 10.1080/23802359.2016.1149789 PMC7871830 33644337

[16] Boguś MI, Kędra E, Bania J, Szczepanik M, Czygier M, Jabłoński P, et al Different defense strategies of *Dendrolimus pini*, *Galleria mellonella*, and *Calliphora vicina* against fungal infection. Journal of Insect Physiology. 2007; 53(9): 909–922. 10.1016/j.jinsphys.2007.02.016 17512001

[17] Arunkumar KP, Metta M, Nagaraju J. Molecular phylogeny of silkmoths reveals the origin of domesticated silkmoth, *Bombyx mori* from Chinese *Bombyx mandarina* and paternal inheritance of *Antheraea proylei* mitochondrial DNA. Molecular Phylogenetics & Evolution. 2006; 40(2):419–427. 10.1016/j.ympev.2006.02.023 16644243

[18] Dai LS, Zhou XD, Kausar S, Abbas MN, Wu L, Zhou HL. Mitochondrial genome of *Diaphania indica* (saunders) (Lepidoptera: Pyraloidea) and implications for its phylogeny. International Journal of Biological Macromolecules. 2017; 108:981–989. 10.1016/j.ijbiomac.2017.11.011 29137996

[19] Yi JQ, Que SQ, Xin TR, Xia B, Zou ZW. Complete mitochondrial genome of *Thitarodes pui* (Lepidoptera: Hepialidae). Mitochondrial DNA. 2016; 27(1): 109–116. 10.3109/19401736.2013.873926 24438300

[20] Kim MJ, Kang AR, Jeong HC, Kim KG, Kim I. Reconstructing intraordinal relationships in Lepidoptera using mitochondrial genome data with the description of two newly sequenced lycaenids, *Spindasis takanonis* and *Protantigius superans* (Lepidoptera: Lycaenidae). Molecular Phylogenetics and Evolution. 2011; 61(2):436–445. 10.1016/j.ympev.2011.07.013 21816227

[21] Kim KG, Hong MY, Kim MJ, Im HH, Kim IM, Bae CH, et al Complete mitochondrial genome sequence of the yellow-spotted long-horned beetle *Psacothea hilaris* (Coleoptera: Cerambycidae) and phylogenetic analysis among Coleopteran insects. Molecules & Cells. 2009; 27(4):429–441. 10.1007/s10059-009-0064-5 19390824

[22] Kikuchi T, Afrin T, Yoshida M. Complete mitochondrial genomes of four entomopathogenic nematode species of the genus *Steinernema*. Parasites & Vectors. 2016; 9(1):430 10.1186/s13071-016-1730-z 27494995 PMC4974692

[23] Li WJ, Wang ZQ, Che YL. The complete mitogenome of the wood-feeding cockroach *Cryptocercus meridianus* (Blattodea: Cryptocercidae) and its phylogenetic relationship among cockroach Families. International Journal of Molecular Sciences. 2017; 18(11):2397 10.3390/ijms18112397 PMC5713365 29137151

[24] Borgstrom E, Lundin S, Lundeberg J. Large scale library generation for high throughput sequencing. PLoS One. 2011; 6: e19119 10.1371/journal.pone.0019119 21589638 PMC3083417

[25] Luo R, Liu B, Xie Y, Li Z, Huang W, Yuan J, et al SOAPdenovo2: an empirically improved memory-efficient short-read de novo assembler. GigaScience. 2012; 1(1):18 10.1186/2047-217X-1-18 23587118 PMC3626529

[26] Lowe TM, Eddy SR. tRNAscan-SE: a program for improved detection of transfer RNA genes in genomic sequence. Nucleic acids research. 1997; 25(5): 0955–964. Available from: https://www.ncbi.nlm.nih.gov/pubmed/9023104/10.1093/nar/25.5.955 PMC146525 9023104

[27] Doose D, Grand C, Lesire C. MAUVE runtime: A component-based middleware to reconfigure software architectures in real-time. Robotic Computing (IRC), IEEE International Conference, Taichung, Taiwan, 10–12 April 2017, IEEE: Taichung, Taiwan, 2017; pp. 208–211. Available from: https://ieeexplore.ieee.org/document/7926540

[28] Stamatakis A, Lin F. Parallel divide-and-conquer phylogeny reconstruction by maximum likelihood. Lecture Notes in Computer Science. 2005; 3726:776–785. Available from: https://www.semanticscholar.org/paper/Parallel-Divide-and-Conquer-Phylogeny-by-Maximum-Du-Stamatakis/62031f2a9cba367422f4acef6d98ef0dd1784f8d

[29] Ronquist F, Teslenko M, Mark PVD. MrBayes 3.2: Efficient bayesian phylogenetic inference and model choice across a large model space. Systematic Biology. 2012; 61(3):539–542. Available from: 10.1093/sysbio/sys029 22357727 PMC3329765

[30] Stewart JB, Beckenbach AT. Insect mitochondrial genomics 2: the complete mitochondrial genome sequence of a giant stonefly, Pteronarcys princes, asymmetric directional mutation bias, and conserved plecopteran A+T-region elements. Genome. 2006; 52(1):815–824. Available from: https://www.ncbi.nlm.nih.gov/pubmed/16936790 10.1139/g06-037 16936790

[31] Ma C, Liu CX, Yang PC, Kang L. The complete mitochondrial genomes of two band-winged grasshoppers, *Gastrimargus marmoratus* and *Oedaleus asiaticus*. BMC Genomics. 2009; 10(1):156 10.1186/1471-2164-10-156 19361334 PMC2674460

[32] Dai LS, Kausar S, Abbas MN, Wang TT. Complete sequence and characterization of the *Ectropis oblique* mitochondrial genome and its phylogenetic implications. International Journal of Biological Macromolecules. 2017; 107:1142–1150. 10.1016/j.ijbiomac.2017.09.093 28962847

[33] Liu YQ, Chen DB, Liu HH, Hu HL, Bian HX, Zhang RS, et al The complete mitochondrial genome of the longhorn beetle *Dorysthenes paradoxus* (Coleoptera: Cerambycidae: Prionini) and the implication for the phylogenetic relationships of the Cerambycidae species. Journal of Insect Science. 2018; 18(2). Available from: https://www.ncbi.nlm.nih.gov/pmc/articles/PMC5833319/10.1093/jisesa/iey012 PMC5833319 29718483

[34] Lu C, Liu YQ, Liao SY, Li B, Xiang ZH, Han H, et al Complete sequence determination and analysis of *Bombyx mori* mitochondrial genome. Journal of Agricultural Biotechnology. 2002; 10(2):163–170. Available from: http://en.cnki.com.cn/Article_en/CJFDTotal-NYSB200202015.htm

[35] Song N, Cai WZ, Li H. Deep-level phylogeny of Cicadomorpha inferred from mitochondrial genomes sequenced by NGS. Scientific Reports. 2017; 7(1):10429 10.1038/s41598-017-11132-0 28874826 PMC5585334

[36] Mccomish BJ, Hills SFK, Biggs PJ, Penny D. Index-free de eovo assembly and deconvolution of mixed mitochondrial genomes. Genome Biology and Evolution. 2010; 2:410–424. Available from: 10.1093/gbe/evq029 20624744 PMC2997550

[37] Gillett CP, Crampton-Platt A, Timmermans MJ, Jordal BH, Emerson BC, Vogler AP. Bulk de novo mitogenome assembly from pooled total DNA elucidates the phylogeny of weevils (Coleoptera: Curculionoidea). Molecular Biology & Evolution. 2014; 31(8):2223–2237. 10.1093/molbev/msu154 24803639 PMC4104315

[38] Saito S, Tamura K, Aotsuka T. Replication origin of mitochondrial DNA in insects. Genetics. 2005; 171(4):1695–1705. 10.1534/genetics.105.046243 16118189 PMC1456096

[39] Moreno M, Marinotti O, Krzywinski J, Tadei WP, James AA, Achee NJ, et al Complete mtDNA genomes of *Anopheles darlingi* and an approach to anopheline divergence time. Malaria Journal. 2010; 9:127 10.1186/1475-2875-9-127 20470395 PMC2877063

[40] Behura SK, Lobo NF, Haas B, Debruyn B, Lovin DD, Shumway MF, et al Complete sequences of mitochondrial genomes of *Aedes aegypti* and *Culex quinquefasciatus* and comparative analysis of mitochondrial DNA fragments inserted in the nuclear genomes. Insect Biochemistry and Molecular Biology. 2011; 41(10):770–777. 10.1016/j.ibmb.2011.05.006 21640823 PMC3162086

[41] Zhou ZJ, Huang Y, Shi FM, Ye HY. The complete mitogenome of *Deracantha onos* (Orthoptera: Bradyporidae). Molecular Biology Reports. 2009; 36(1):7–12. 10.1007/s11033-007-9145-8 17891510

[42] Dai QY, Gao Q, Wu CS., Chesters D, Zhu CD, Zhang AB. Phylogenetic reconstruction and DNA barcoding for closely related pine moth species (*Dendrolimus*) in China with multiple gene markers. PLoS One. 2012; 7(4):e32544 10.1371/journal.pone.0032544 22509245 PMC3317921

[43] Sun Y, Chen C, Gao J, Abbas MN, Kausar S, Qian C, et al Comparative mitochondrial genome analysis of *Daphnis nerii* and other Lepidopteran insects reveals conserved mitochondrial genome organization and phylogenetic relationships. PLoS One. 2017; 12(6): e0178773 10.1371/journal.pone.0178773 28598968 PMC5466310

[44] Stewart JB, Beckenbach AT. Insect mitochondrial genomics: the complete mitochondrial genome sequence of the meadow spittlebug *Philaenus spumarius* (Hemiptera: Auchenorrhyncha: Cercopoidae). Genome. 2005; 48(1):46–54. 10.1139/g04-090 15729396

[45] Jost MC, Shaw KL. Phylogeny of *Ensifera* (Hexapoda: Orthoptera) using three ribosomal loci, with implications for the evolution of acoustic communication. Mol Phylogenet Evol. 2006; 38(2):510–530. 10.1016/j.ympev.2005.10.004 16298145

[46] Yamauchi MM, Miya MU, Nishida M. Use of a PCR-based approach for sequencing whole mitochondrial genomes of insects: two examples (cockroach and dragonfly) based on the method developed for decapod crustaceans. Insect Mol Biol. 2004; 13(4):435–442. 10.1111/j.0962-1075.2004.00505.x 15271216

[47] Carapelli A, Liò P, Nardi F, van der Wath E, Frati F. Phylogenetic analysis of mitochondrial protein coding genes confirms the reciprocal paraphyly of Hexapoda and Crustacea. BMC Evol Biol. 2007; 7 (Suppl 2): S8 10.1186/1471-2148-7-S2-S8 PMC1963475 17767736

[48] Botero-Castro F, Tilak MK, Justy F, Catzeflis F, Delsuc F, Douzery EJP. Next-generation sequencing and phylogenetic signal of complete mitochondrial genomes for resolving the evolutionary history of leaf-nosed bats (Phyllostomidae). Mol Phylogenet Evol. 2013; 69(3):728–739. 10.1016/j.ympev.2013.07.003 23850499

[49] Bernt M, Bleidorn C, Braband A, Dambach J, Donath A, Fritzsch G, et al A comprehensive analysis of bilateran mitochondrial genomes and phylogeny. Mol Phylogenet Evol. 2013; 69(2):352–364. 10.1016/j.ympev.2013.05.002 23684911

[50] Cameron SL, Lo N, Bourguignon T, Svenson GJ, Evans TA. A mitochondrial genome phylogeny of termites (Blattodea: Termitoidae): robust support for interfamilial relationships and molecular synapomorphies define major clades. Mol Phylogenet Evol. 2012; 65(1):163–173. 10.1016/j.ympev.2012.05.034 22683563

[51] Pacheco MA, Battistuzzi FU, Lentino M, Aguilar RF, Kumar S, Escalante AA. Evolution of modern birds revealed by mitogenomics: timing the radiation and origin of major orders. Mol Biol Evol. 2011; 28(6):1927–1942. 10.1093/molbev/msr014 21242529 PMC3144022

[52] Wu LY. Characterization of the complete mitochondrial genomes of *Euthrix laeta* and *Trabala vishnou guttata* and phylogenetic analyses. M.Sc. Thesis, Nanchang University. 2017. Available from: https://kns.cnki.net/KCMS/detail/detail.aspx?dbcode=CMFD&dbname=CMFD201801&filename=1017233049.nh&v=MDM1NDIyNkdiRzdIZEhJcHBFYlBJUjhlWDFMdXhZUzdEaDFUM3FUcldNMUZyQ1VSN3FmWk9SdEZ5amtXcnZOVkY=

[53] Wang XY, Zhou LH. Genetic diversity and population history among geographic populations of *Spodoptera exigua* in North China based on mtDNA Cytb gene sequences. Acta Ecologica Sinica. 2016; 36(8). Available from: http://www.ecologica.cn/stxb/ch/reader/view_abstract.aspx?file_no = stxb201410041952&flag = 1

[54] Kim MJ, Wang AR, Park JS, Kim I. Complete mitochondrial genomes of five skippers (Lepidoptera: Hesperiidae) and phylogenetic reconstruction of Lepidoptera. Gene. 2014; 549:97–112. 10.1016/j.gene.2014.07.052 25058696

[55] Park JS, Kim MJ, Jeong SY, Kim SS, Kim I. Complete mitochondrial genomes of two gelechioids, *Mesophleps albilinella* and *Dichomeris ustalella* (Lepidoptera: Gelechiidae), with a description of gene rearrangement in Lepidoptera. Curr Genet. 2016; 62:809–826. 10.1007/s00294-016-0585-3 26952721

[56] Cao YQ, Ma CA, Chen JY, Yang DR. The complete mitochondrial genomes of two ghost moths, *Thitarodes renzhiensis* and *Thitarodes yunnanensis*: the ancestral gene arrangement in Lepidoptera. BMC genomics. 2012; 13(1):276 10.1186/1471-2164-13-276 22726496 PMC3463433

