# Supplementary figures and images for: Comparative mitochondrial genome analysis of Dendrolimus houi (Lepidoptera: Lasiocampidae) and phylogenetic relationship among Lasiocampidae species
Source: PLoS One. 2020 May 14;15(5):e0232527. doi: 10.1371/journal.pone.0232527 (PMC7224488; doi:10.1371/journal.pone.0232527)

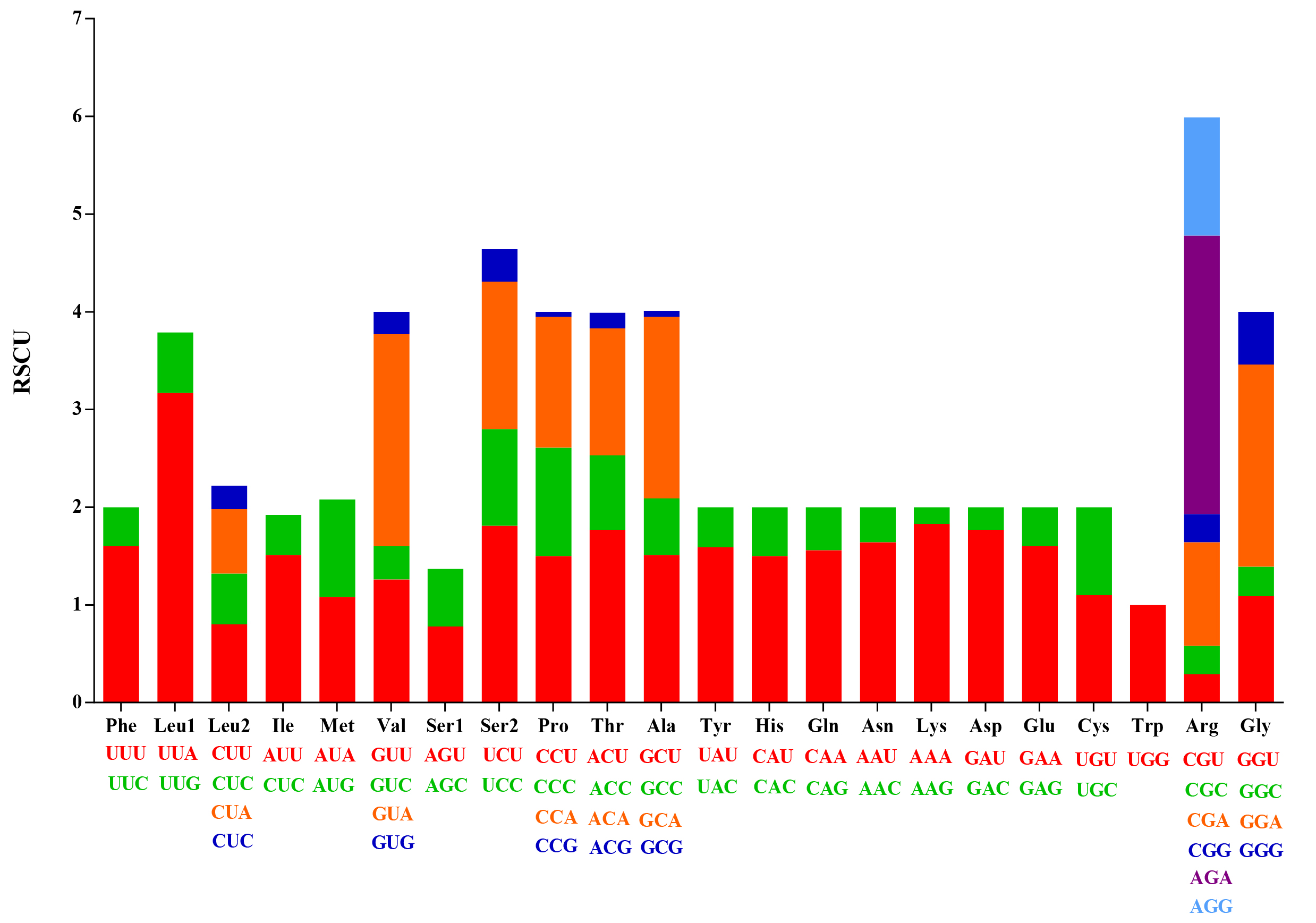

Supplement: S1 Fig — (TIF) [file pone.0232527.s001.tif]

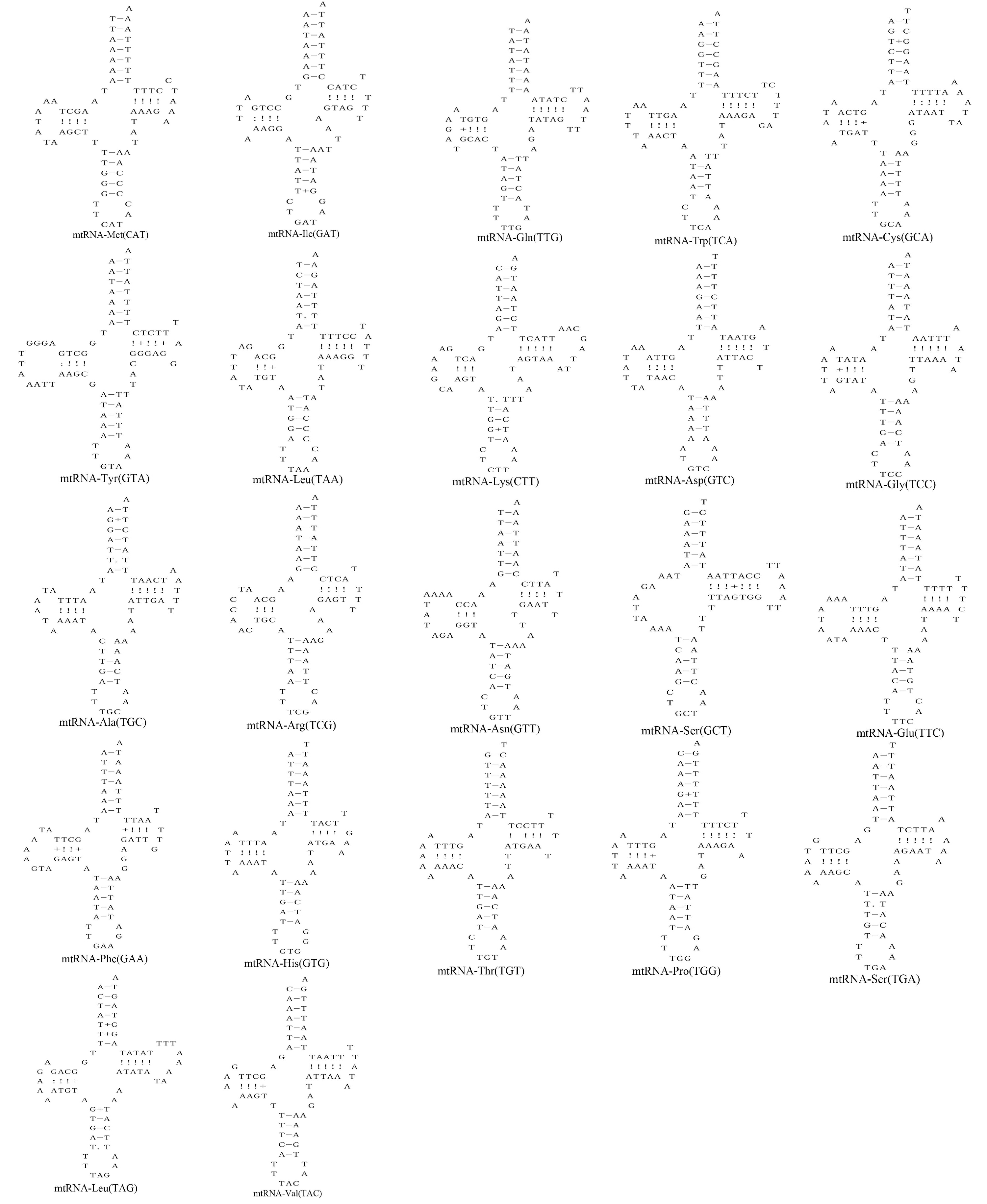

Supplement: S2 Fig — (TIF) [file pone.0232527.s002.tif]

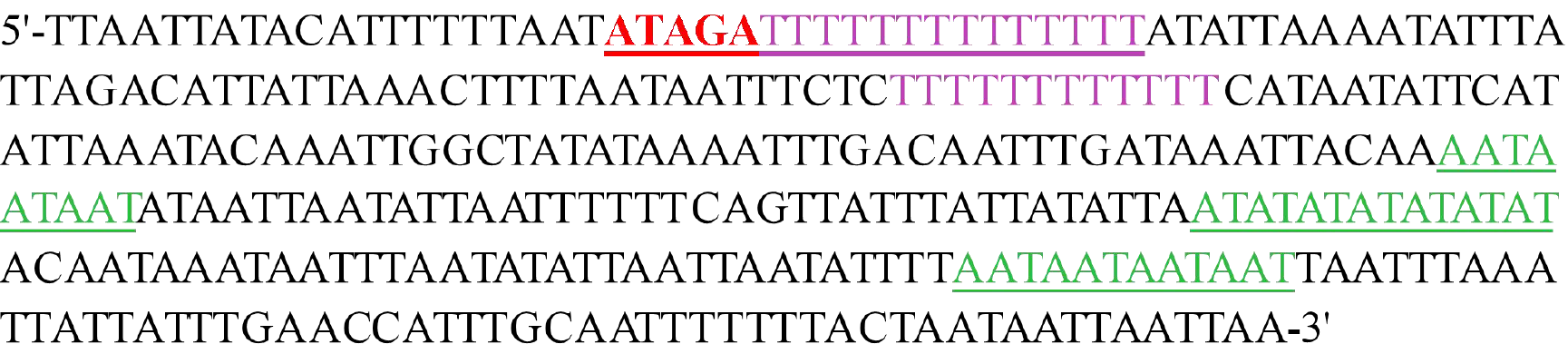

Supplement: S3 Fig — (TIF) [file pone.0232527.s003.tif]
